# Involuntary displacement and self-reported health in a cross-sectional survey of people experiencing homelessness in Denver, Colorado, 2018–2019

**DOI:** 10.1186/s12889-024-18681-w

**Published:** 2024-04-25

**Authors:** Ashley A Meehan, Katherine E Milazzo, Michael Bien, Samantha K Nall, Katherine Diaz Vickery, Emily Mosites, Joshua A Barocas

**Affiliations:** 1 Department of Health, Behavior, and Society, Johns Hopkins Bloomberg School of Public Health, 624 N. Broadway, Baltimore, MD 21205 USA; 2 Our House Georgia, Atlanta, GA USA; 3 National Foundation for the Centers for Disease Control and Prevention, Atlanta, GA USA; 4 Divisions of General Internal Medicine and Infectious Diseases, University of Colorado School of Medicine, Aurora, CO USA; 5 Health, Homelessness, & Criminal Justice Lab, Hennepin Health Care Research Institute, Minneapolis, MN USA; 6 Office of Readiness and Response, Centers for Disease Control and Prevention, Atlanta, GA USA

**Keywords:** Homelessness, Involuntary displacement, Health equity

## Abstract

**Background:**

The number of people experiencing unsheltered homelessness in the U.S. is increasing. Municipalities have responded with punitive responses such as involuntary displacement (i.e., encampment sweeps, move along orders), but little is known about the impact of involuntary displacement on health. The purpose of this study was to investigate the association between broadly defined experiences of involuntary displacement and self-reported health conditions among people experiencing homelessness.

**Methods:**

We used logistic regression models to generate odds ratios using publicly available data from a cross-sectional sample of people experiencing homelessness in Denver, Colorado, during September 2018-February 2019. Hosmer-Lemeshow Goodness of Fit tests were used to assess model fit.

**Results:**

Among 397 people experiencing homelessness, involuntary displacement was significantly associated with self-reported infectious diseases (adjusted odds ratio (aOR) 2.09, 95% CI 1.27, 3.41), substance and alcohol use (aOR 2.83; 95% CI 1.70, 4.73), climate-related conditions (aOR 2.27; 95% CI 1.35, 3.83), and worsening mental health (aOR 2.00; 95% CI 1.24, 3.24) after controlling for potential confounders. No statistically significant associations were identified between involuntary displacement and injuries, musculoskeletal issues, chronic conditions, and chronic mental and emotional issues.

**Conclusions:**

This research quantifies the association between involuntary displacement and multiple infectious and non-infectious health outcomes. While city officials attempt to grapple with increasing unsheltered homelessness, it is important to understand what harms are occurring that are associated with current policies. Our research adds to the growing body of literature that involuntary displacement is a harmful response to unsheltered homelessness. Alternative approaches focused on connections to housing and social services should be prioritized.

**Supplementary Information:**

The online version contains supplementary material available at 10.1186/s12889-024-18681-w.

## Background

From 2020 to 2022, the number of people experiencing unsheltered homelessness in the U.S. increased by 3.4% [1]. By 2022, 40% of the 582,500 people experiencing homelessness were unsheltered, meaning they slept outside, in a car, abandoned building, or other place not meant for human habitation [1]. While people experiencing homelessness in the U.S. bear an inequitable burden of infectious and non-infectious diseases compared to their housed counterparts [2–5], research has shown that unsheltered homelessness brings different health risks compared to sheltered homelessness. Unsheltered homelessness is associated with higher rates of mental illness, substance use, injuries, and other physical illnesses compared to sheltered homelessness [6]. In reality, many people move between sheltered and unsheltered homelessness (i.e., sleeping in a shelter some nights and sleeping outside other nights) [7]. During times when they are unsheltered, people are at risk for involuntary displacement [8, 9]. 

Involuntary displacement of people experiencing homelessness occurs when local law enforcement or other government officials force people to move from a location, and can take shape as encampment “clearings,” “sweeps,” or being “moved along.” [10–12] Acts of displacement may be conducted in response to local ordinances regarding camping, loitering, trespassing, or perceived nuisance, and often results in the destruction of temporary outdoor shelters and confiscation of personal belongings [12]. Involuntary displacements of people experiencing homelessness are sometimes accompanied by connections to housing and with social service supports, but often not. As an illustration, in Denver, Colorado – where 30% of the people experiencing homelessness are unsheltered [13] – city officials enacted a “camping ban” in 2012, which allows involuntary displacement of people experiencing unsheltered homelessness [14]. However, scientific literature on the health impacts of involuntary displacement is limited [12, 15, 16]. 

The health impacts of involuntary displacement have been largely explored in the context of substance use; in previous studies, people experiencing displacement had lower odds of being in substance use treatment, may be more likely to share injection drug use equipment, and are likely to experience increased morbidity and mortality due to injection drug use [15, 16]. However, existing research on involuntary displacement also shows that people who are displaced lose medications and survival necessities (including identification cards and telephones), are disconnected from social support networks, and may be pushed further into covert locations [12, 17]. When people are forced to disperse or involuntarily relocate, they may face additional distance and transportation barriers to accessing health care, which can exacerbate existing conditions [18, 19]. Additionally, losing essential survival items such as blankets, cooling fans, or tarps can change exposure to the environment and lead to climate-related conditions like frostbite or heat stroke [20, 21]. In light of these potential outcomes after displacement, we aimed to investigate the association between involuntary displacement and various health outcomes in a cross-sectional survey of people experiencing homelessness in Denver, Colorado.

## Methods

### Data source and study design

We conducted a retrospective analysis using publicly available cross-sectional survey data from 484 participants in Denver, Colorado, between September 2018 and February 2019 [22]. An in-depth description of the original study including data collection methods and data collection tools has been described elsewhere [23]. Briefly, participants were all people experiencing homelessness accessing services at select sites around Denver (e.g., homeless shelters, soup kitchens, day service providers, street corners, homeless encampments, and immigrant day laborer centers). Within each of these sites, surveys were conducted at multiple points throughout the day with convenience samples of people at the site to understand experiences with law enforcement and self-reported health and safety.

We accessed the publicly available dataset via the Inter-University Consortium for Political and Social Research in March 2023 [22]. This research is exempt from human subjects review according to specifications of secondary research of de-identified data in 45 CFR 46.104 [24]. 

### Measures

The primary exposure of interest was involuntary displacement, defined as being “moved along” by law enforcement one or more times in the previous twelve months. Participants were asked: “*In the last year, how many times, if any, have enforcement individuals requested/required that you ‘move along’ from your current location or activity? Never, one time, 2–5 times, 6–10 times, more than 10 times*.” The exposure was recoded as a binary variable: never displaced or displaced one or more times. While involuntary displacement is typically conceptualized as involving loss of belongings and disrupted living environment, this measure also captured loitering in public places and other experiences of being moved-along without loss of possessions or sleeping location.

Four questions were used to generate eight binary health outcome variables: (1) From a list of pre-specified health issues: “*Do you experience any of the following significant physical health problems? Check all that apply*;” (2) “*Do you experience significant mental or emotional health problems? Yes or no*;” (3) “*Have you ever been diagnosed with a mental health or emotional issue? Yes or no*;” and (4) “*How would you describe your overall mental health in the last year? I feel better now than a year ago, I feel about the same now as I have felt most of the year, or I am feeling much worse than about a year ago*.” Health issues were grouped into eight categories: infectious diseases (influenza or pneumonia, frequent bacterial infections, hepatitis C, HIV, tuberculosis); substance and alcohol use and misuse; climate-related health conditions (dehydration, frostbite, heat stroke); injuries (untreated injury, traumatic brain injury, wounds that won’t heal); musculoskeletal issues and other mobility disabilities (joint pain, back pain, other physical disability); chronic health conditions (cancer, diabetes, heart problems); chronic mental and emotional conditions; and acute worsening of mental health (Table 1). We included age, race and ethnicity, gender identity, duration of homelessness, and primary sleeping setting as covariates. Primary sleeping setting was defined as unsheltered or sheltered.

**Table 1:** Self-reported health by displacement status among people experiencing homelessness in Denver, Colorado, September 2018-February 2019

Outcome	Overall study sample *N* = 397	Never displaced *N* = 159	Displaced one or more times *N* = 238
Infectious Diseases	115 (29%)	33 (21%)	82 (35%)
Frequent Bacterial Infections	44 (11%)	12 (8%)	32 (13%)
HIV/AIDS	11 (3%)	4 (3%)	7 (3%)
Tuberculosis	4 (1%)	3 (2%)	1 (0.4%)
Recent Flu or Pneumonia	49 (12%)	13 (8%)	36 (15%)
Hepatitis C	39 (10%)	9 (6%)	30 (13%)
Substance and Alcohol Use Disorder	132 (33%)	30 (19%)	102 (43%)
Climate-Related Outcomes	109 (28%)	29 (18%)	80 (34%)
Frostbite & Related Sequelae	40 (10%)	9 (6%)	31 (13%)
Dehydration	79 (20%)	16 (10%)	63 (27%)
Heat Stroke	24 (6%)	10 (6%)	14 (6%)
Injury Outcomes	90 (23%)	27 (17%)	63 (27%)
Untreated Significant Injury	33 (8%)	7 (4%)	26 (11%)
Traumatic Brain Injury	53 (13%)	19 (12%)	34 (14%)
Wound that won’t heal	31 (8%)	5 (3%)	26 (11%)
Chronic Outcomes	83 (21%)	24 (21%)	49 (21%)
Heart Problems	43 (11%)	16 (10%)	27 (11%)
Cancer	19 (5%)	6 (4%)	13 (6%)
Diabetes	34 (9%)	13 (8%)	21 (9%)
Musculoskeletal & Disability Outcomes	235 (59%)	86 (54%)	149 (63%)
Joint Pain	121 (31%)	41 (26%)	80 (34%)
Back Pain	157 (40%)	52 (33%)	105 (44%)
Other Physical Disability	119 (30%)	43 (27%)	76 (32%)
Mental and Emotional Outcomes	271 (68%)	112 (70%)	159 (67%)
Post-Traumatic Stress Disorder	111 (28%)	35 (22%)	76 (32%)
Experiences a Mental or Emotional Problem	156 (39%)	77 (48%)	79 (33%)
Has received a Mental or Emotional Diagnosis	153 (39%)	78 (50%)	75 (32%)
Worsening Mental Health in Past Year	139 (35%)	41 (28%)	98 (43%)
Missing Change in Mental Health	19 (5%)	10 (6%)	9 (4%)

### Analytic approach

We performed this analysis using Stata v.17 (StataCorp. 2021. *Stata Statistical Software: Release* 17. College Station, TX: StataCorp LLC.). We did not identify any patterns or systematic differences in missingness, so observations missing information on the exposure, the outcomes, or the covariates of interest were excluded in this analysis. A complete case analysis was conducted using 397 participant observations. We generated unadjusted and adjusted logistic regression models for each of the eight binary health outcomes to estimate odds ratios. Unadjusted models estimated the odds ratio of each binary health outcome by the binary exposure of involuntary displacement. Adjusted multivariable models additionally included age, race and ethnicity, gender identity, duration of homelessness, and primary sleeping setting as categorical covariates in the logistic regression models. Covariates included as potential confounders were determined using a Directed Acyclic Graph (DAG) [25]. 

## Results

Among the analytic sample of 397 participants, most were aged 45 or older (51%), identified as non-Hispanic white (41%), and as cisgender males (68%) (Table 2). Over half of participants (55%) had been experiencing homelessness for two or more years at the time of the survey and were primarily sleeping outside, in a car, or in an abandoned building (67%). Participants who reported involuntary displacement were more likely to have been experiencing homelessness for a longer duration (*p* = 0.02) and more likely to have been experiencing unsheltered homelessness compared to sheltered homelessness (*p* < 0.001). The prevalence of self-reported health outcomes can be found in Table 1.

**Table 2:** Characteristics of people experiencing homelessness by displacement status in Denver, Colorado, September 2018-February 2019

Characteristic	Overall study sample^1^ *N* = 397	Never displaced *N* = 159	Displaced one or more times *N* = 238	Fisher’s exact chi square *p*-value
Age				0.29
Under 24^2^	38 (10%)	14 (9%)	24 (10%)	
25–34	80 (20%)	27 (17%)	53 (22%)	
35–44	79 (20%)	29 (18%)	50 (21%)	
45–54	102 (26%)	41 (26%)	61 (26%)	
55+	98 (25%)	48 (30%)	50 (21%)	
Race and Ethnicity				0.13
Non-Hispanic White	163 (41%)	62 (39%)	101 (42%)	
Black or African American	59 (15%)	25 (16%)	34 (14%)	
Hispanic or Latinx	70 (18%)	36 (23%)	34 (14%)	
Mixed Race or Other^3^	105 (26%)	36 (23%)	69 (29%)	
Gender Identity				0.22
Cisgender Male	269 (68%)	100 (63%)	169 (71%)	
Cisgender Female	110 (28%)	51 (32%)	59 (25%)	
Transgender, Nonbinary, or Other^4^	18 (5%)	8 (5%)	10 (4%)	
Duration Homeless				0.02*
< 6 months	43 (11%)	26 (16%)	17 (7%)	
6–12 months	44 (11%)	20 (13%)	24 (10%)	
1–2 years	92 (23%)	37 (23%)	55 (23%)	
> 2 years	218 (55%)	76 (48%)	142 (60%)	
Primary Sleeping Setting				< 0.001*
Sheltered or other	133 (34%)	81 (51%)	52 (22%)	
Outside, in a car, or abandoned building	264 (67%)	78 (49%)	186 (78%)	

In the unadjusted analyses, we found that, compared to people who had not experienced involuntary displacement, people who experienced displacement had statistically significant higher odds of experiencing infectious diseases (odds ratio [OR] 2.01; 95% CI 1.26, 3.20), substance and alcohol use (OR 3.23; 95% CI 2.01, 5.18), climate-related outcomes (OR 2.27; 95% CI 1.40, 3.68), injuries (OR 1.76; 95% CI 1.06, 2.91), and worsening mental health outcomes (OR 1.97; 95% CI 1.26, 3.07) (Table 3).

**Table 3:** Association between involuntary displacement and health outcomes among a cross-sectional sample of people experiencing homelessness

Health Outcome Category	Unadjusted^5^ OR (95% CI)	Adjusted^6^ OR (95% CI)
Infectious Diseases	2.01 (1.26, 3.20)*	2.09 (1.27, 3.41)*
Substance and Alcohol Use Disorders	3.23 (2.01, 5.18)*	2.83 (1.70, 4.73)*
Climate-Related Outcomes	2.27 (1.40, 3.68)*	2.27 (1.35, 3.83)*
Injury-Related Outcomes	1.76 (1.06, 2.91)*	1.35 (0.78, 2.31)
Chronic Health Outcomes	0.95 (0.58, 1.56)	1.08 (0.63, 1.86)
Musculoskeletal and Disability Outcomes	1.42 (0.95, 2.14)	1.33 (0.85, 2.08)
Mental and Emotional Health Outcomes	0.84 (0.55, 1.30)	0.89 (0.56, 1.43)
Worsening Mental Health in the Past Year^7^	1.97 (1.26, 3.07)*	2.00 (1.24, 3.24)*

After controlling for age, race and ethnicity, gender identity, duration homeless, and primary outdoor sleeping setting, the relationships were not qualitatively changed for infectious diseases (adjusted odds ratio [aOR] 2.09; 95% CI 1.27, 3.41), substance and alcohol use (aOR 2.83; 95% CI 1.70, 4.73), climate-related outcomes (aOR 2.27; 95% CI 1.35, 3.83), and worsening mental health (aOR 2.00; 95% CI 1.24, 3.24). The addition of possible confounders attenuated the relationship between involuntary displacement and injuries and the relationship was no longer statistically significant (aOR 1.35; 95% CI 0.78, 2.31).

There were no statistically significant associations between involuntary displacement and chronic health outcomes, musculoskeletal issues and other disabilities, and chronic mental and emotional issues in the unadjusted or adjusted models. For all adjusted models, the Hosmer-Lemeshow Goodness of Fit test yielded *p*-values greater than 0.05 (range: 0.13–0.40), indicating that the models fit the data well [26]. Supplemental Tables 1–8 provide the unadjusted and adjusted odds ratios for each of the eight health outcomes.

## Discussion

This analysis showed that, among a cross-sectional sample of people experiencing homelessness in Denver, Colorado, involuntary displacement was associated with self-reported infectious diseases, substance and alcohol use, climate-related outcomes, and worsening mental health after controlling for several relevant covariates. These findings quantify the association between involuntary displacement and infectious and non-infectious health outcomes among people experiencing homelessness in Denver.

Because responses to unsheltered homelessness occur at local levels, acts of displacement (i.e., encampment sweeps) differ across jurisdictions. Encampment responses that are human-centered and prioritize housing, connections to care and services, and support client needs may not lead to similar outcomes when compared with encampment responses that result in loss of belongings and disconnection from care. As such, further research is needed to identify the various social and behavioral mechanisms through which involuntary displacement influences new or worsening health outcomes, and how these mechanisms and outcomes differ across varying encampment closure experiences. Longitudinal studies and robust observational research designs could further isolate the effect of involuntary displacement on the health of people experiencing homelessness.

Further, experiencing displacement may be correlated with duration of homelessness. We found a statistically significant difference in reported duration of homelessness between those that reported involuntary displacement in the previous twelve months and those that did not report involuntary displacement. After creating a DAG, we identified duration of homelessness as a confounder of the relationship between involuntary displacement and health outcomes. As such, we have included duration of homelessness as a covariate in our models to control for duration of homelessness. Because of this analytic decision, we feel that we have appropriately ensured that duration of homelessness did not affect our estimates in our adjusted models.

This analysis is subject to limitations. First, we cannot determine temporality or causality in the relationship between involuntary displacement and health outcomes because of the cross-sectional design. However, it is intuitive that injuries such as frostbite are only likely to occur after confiscation of items like blankets and heaters, not before. Second, asking participants at one point in time to recall how often they have been moved along by law enforcement over the past twelve months may introduce recall bias. However, creating a binary indicator of being moved along likely addressed this potential limitation, since people who have been moved along likely reported being moved along at least once, even if they did not remember the exact number of times they had been moved along. Third, conducting secondary analysis on existing data limits our ability to include additional covariates that may influence or confound the relationship between involuntary displacement and health conditions. Specifically, we were not able to control for how social networks and experiences of stigma and discrimination influence health outcomes after experiencing displacement.

We also were unable to control for access to health care services, which may be challenged when experiencing homelessness and even more precarious after experiencing displacement. Fourth, we were not able to determine if there were intended or unintended positive effects of being displaced such as moving closer to a methadone clinic or medical clinic. Finally, variations in what involuntary displacement entails can have variable impacts on different health conditions but this nuance was not explored here. In some instances, people may be forced to disperse from commercial areas to minimize daytime loitering, while others may lose access to all their belongings and have no place to sleep. Unfortunately, we were not able to distinguish the spectrum of displacement experiences in this analysis. Qualitative and quantitative studies to further refine conceptualization and definitions of involuntary displacement of people experiencing homelessness could advance research aiming to understand how different experiences of displacement impact health. Nevertheless, there is general consensus from previous studies that involuntary displacement when not coupled with wrap around services is more commonly disruptive and damaging to an individual than helpful or uplifting [12]. 

Considering these and other recent findings, evaluating current displacement policies and exploring alternative approaches that center the health needs of people experiencing homeless are important. Numerous strategies have been identified from communities across the U.S. to reduce the harms of involuntary displacement: provide trainings and incentives for law enforcement to utilize diversion pathways and limit criminalization; leverage existing funds and resources to maximize crisis response services, mental health, and substance use services; improving and expanding existing shelter options; and finally, prioritizing housing and ensuring people are connected to social support services to receive necessary housing supports [27–32]. 

## Conclusion

In this analysis, involuntary displacement was statistically significantly associated with worsening acute health conditions among people experiencing homelessness. While city officials attempt to grapple with increasing numbers of people experiencing homelessness, it is important for them to understand what harms are occurring that are associated with current policies. While “housing for all” is a laudable goal, such infrastructure takes time. Our analysis adds to the growing body of literature that suggests that the “in the meantime” approach of displacing people is harmful and, ultimately, worsens public health.

### Electronic supplementary material

Below is the link to the electronic supplementary material.

Supplementary Material 1

## Data Availability

The dataset analyzed during the current study is available in the Inter-University Consortium for Political and Social Research repository, https://www.openicpsr.org/openicpsr/project/112202/version/V1/view.

## Footnotes

1 Column percentages may not equal 100 exactly due to rounding.

2 Age category under 18 and 18–24 were collapsed due to small cell sizes.

3 Asian, American Indian, Hawaiian/Pacific Islander, and Mixed/Other were collapsed due to small cell sizes.

4 Transgender male, transgender female, and non-binary or other were collapsed due to small cell sizes.

5 Unadjusted models present the relationship between the exposure and the outcome only.

6 Adjusted models included age, race and ethnicity, gender, duration homeless, and primary sleeping setting as confounders.

7 19 of the 397 observations were missing values for change in mental health over the past twelve months, bringing the total observations used to evaluate worsening mental health and being displaced to 378. All other models were generated using the sample of 397 observations.
