# Supplementary material for: Involuntary displacement and self-reported health in a cross-sectional survey of people experiencing homelessness in Denver, Colorado, 2018–2019
Source: BMC Public Health. 2024 Apr 25;24:1159. doi: 10.1186/s12889-024-18681-w (PMC11044435; doi:10.1186/s12889-024-18681-w)
Supplement: Supplementary file 1 — Supplementary Material 1 [file 12889_2024_18681_MOESM1_ESM.docx]

**Supplemental Table 1. Unadjusted and adjusted odds ratios of the association between involuntary displacement and infectious diseases among a cross-sectional sample of people experiencing homelessness in Denver, Colorado, September 2018-February 2019.**

| **Characteristic** | | **Unadjusted OR (95% CI)** | **Adjusted OR (95% CI)** |
| --- | --- | --- | --- |
| Involuntary Displacement in Past 12 Months | |  |  |
|  | Never | Ref | Ref |
|  | 1 or more times | 2.01 (1.26, 3.20)* | 2.02 (1.21, 3.35)* |
| Age | |  |  |
|  | Under 24 | Ref | Ref |
|  | 25-34 | 1.93 (0.81, 4.63) | 1.81 (0.73, 4.47) |
|  | 35-44 | 1.49 (0.62, 3.62) | 1.33 (0.53, 3.35) |
|  | 45-54 | 0.79 (0.32, 1.92) | 0.78 (0.31, 1.98) |
|  | 55+ | 1.49 (0.63, 3.53) | 1.58 (0.63, 3.96) |
| Race and Ethnicity | |  |  |
|  | Non-Hispanic White | Ref | Ref |
|  | Black or African American | 0.55 (0.28, 1.11) | 0.59 (0.29, 1.22) |
|  | Hispanic or Latinx | 0.96 (0.53, 1.74) | 1.03 (0.55, 1.93) |
|  | Mixed Race or Other | 0.58 (0.33, 1.02) | 0.56 (0.31, 1.00) |
| Gender Identity | |  |  |
|  | Cisgender Male | Ref | Ref |
|  | Cisgender Female | 1.12 (0.69, 1.81) | 1.30 (0.77, 2.17) |
|  | Transgender, Nonbinary, or Other | 0.71 (0.23, 2.23) | 0.86 (0.25, 2.97) |
| Duration Homeless | |  |  |
|  | <6 months | Ref | Ref |
|  | 6-12 months | 3.52 (1.22, 10.16)* | 3.05 (1.01, 9.15)* |
|  | 1-2 years | 2.84 (1.08, 7.48)* | 2.29 (0.83, 6.36) |
|  | >2 years | 2.56 (1.03, 6.37)* | 2.01 (0.76, 5.28) |
| Primary Sleeping Setting | |  |  |
|  | Sheltered or other | Ref | Ref |
|  | Outside, in a car, or an abandoned building | 1.45 (0.90, 2.33) | 1.15 (0.68, 1.96) |

** indicates statistical significance at the alpha=0.05 level.*

**Supplemental Table 2. Unadjusted and adjusted odds ratios of the association between involuntary displacement and substance and alcohol use disorders among a cross-sectional sample of people experiencing homelessness in Denver, Colorado, September 2018-February 2019.**

| **Characteristic** | | **Unadjusted OR (95% CI)** | **Adjusted OR (95% CI)** |
| --- | --- | --- | --- |
| Involuntary Displacement in Past 12 Months | |  |  |
|  | Never | Ref | Ref |
|  | 1 or more times | 3.23 (2.01, 5.18)* | 2.83 (1.70, 4.73)* |
| Age | |  |  |
|  | Under 24 | Ref | Ref |
|  | 25-34 | 2.26 (0.95, 5.40) | 2.24 (0.88, 5.69) |
|  | 35-44 | 2.31 (0.97, 5.52) | 2.39 (0.93, 6.13) |
|  | 45-54 | 1.34 (0.57, 3.17) | 1.34 (0.53, 3.37) |
|  | 55+ | 1.23 (0.51, 2.92) | 1.30 (0.50, 3.35) |
| Race and Ethnicity | |  |  |
|  | Non-Hispanic White | Ref | Ref |
|  | Black or African American | 1.42 (0.76, 2.66) | 1.95 (0.98, 3.85) |
|  | Hispanic or Latinx | 1.50 (0.84, 2.71) | 2.13 (1.12, 4.07)* |
|  | Mixed Race or Other | 1.20 (0.71, 2.03) | 1.28 (0.73, 2.25) |
| Gender Identity | |  |  |
|  | Cisgender Male | Ref | Ref |
|  | Cisgender Female | 0.68 (0.42, 1.11) | 0.84 (0.50, 1.42) |
|  | Transgender, Nonbinary, or Other | 0.35 (0.10, 1.24) | 0.35 (0.09, 1.37) |
| Duration Homeless | |  |  |
|  | <6 months | Ref | Ref |
|  | 6-12 months | 2.59 (0.88, 7.60) | 1.72 (0.55, 5.36) |
|  | 1-2 years | 3.45 (1.32, 9.03)* | 2.15 (0.77, 5.98) |
|  | >2 years | 3.57 (1.45, 8.84)* | 2.23 (0.85, 5.89) |
| Primary Sleeping Setting | |  |  |
|  | Sheltered or other | Ref | Ref |
|  | Outside, in a car, or an abandoned building | 2.29 (1.42, 3.71)* | 1.61 (0.95, 2.74) |

** indicates statistical significance at the alpha=0.05 level.*

**Supplemental Table 3. Unadjusted and adjusted odds ratios of the association between involuntary displacement and climate-related outcomes among a cross-sectional sample of people experiencing homelessness in Denver, Colorado, September 2018-February 2019.**

| **Characteristic** | | **Unadjusted OR (95% CI)** | **Adjusted OR (95% CI)** |
| --- | --- | --- | --- |
| Involuntary Displacement in Past 12 Months | |  |  |
|  | Never | Ref | Ref |
|  | 1 or more times | 2.27 (1.40, 3.68)* | 2.28 (1.35, 3.83)* |
| Age | |  |  |
|  | Under 24 | Ref | Ref |
|  | 25-34 | 0.71 (0.30, 1.71) | 0.69 (0.28, 1.72) |
|  | 35-44 | 1.14 (0.49, 2.65) | 1.23 (0.50, 3.03) |
|  | 45-54 | 0.88 (0.39, 2.02) | 0.92 (0.39, 2.20) |
|  | 55+ | 0.98 (0.43, 2.24) | 1.19 (0.49, 2.91) |
| Race and Ethnicity | |  |  |
|  | Non-Hispanic White | Ref | Ref |
|  | Black or African American | 0.67 (0.33, 1.38) | 0.64 (0.30, 1.35) |
|  | Hispanic or Latinx | 0.72 (0.38, 1.39) | 0.79 (0.39, 1.56) |
|  | Mixed Race or Other | 1.43 (0.84, 2.42) | 1.41 (0.82, 2.44) |
| Gender Identity | |  |  |
|  | Cisgender Male | Ref | Ref |
|  | Cisgender Female | 1.03 (0.62, 1.69) | 1.04 (0.61, 1.76) |
|  | Transgender, Nonbinary, or Other | 1.74 (0.65, 4.66) | 1.89 (0.65, 5.45) |
| Duration Homeless | |  |  |
|  | <6 months | Ref | Ref |
|  | 6-12 months | 0.97 (0.38, 2.42) | 0.86 (0.32, 2.28) |
|  | 1-2 years | 0.68 (0.30, 1.54) | 0.59 (0.24, 1.44) |
|  | >2 years | 0.92 (0.45, 1.87) | 0.69 (0.31, 1.53) |
| Primary Sleeping Setting | |  |  |
|  | Sheltered or other | Ref | Ref |
|  | Outside, in a car, or an abandoned building | 1.38 (0.85, 2.23) | 1.21 (0.71, 2.06) |

** indicates statistical significance at the alpha=0.05 level.*

**Supplemental Table 4. Unadjusted and adjusted odds ratios of the association between involuntary displacement and injury-related outcomes among a cross-sectional sample of people experiencing homelessness in Denver, Colorado, September 2018-February 2019.**

| **Characteristic** | | **Unadjusted OR (95% CI)** | **Adjusted OR (95% CI)** |
| --- | --- | --- | --- |
| Involuntary Displacement in Past 12 Months | |  |  |
|  | Never | Ref | Ref |
|  | 1 or more times | 1.76 (1.06, 2.91)* | 1.35 (0.78, 2.31) |
| Age | |  |  |
|  | Under 24 | Ref | Ref |
|  | 25-34 | 1.58 (0.60, 4.11) | 1.37 (0.51, 3.71) |
|  | 35-44 | 1.71 (0.66, 4.45) | 1.62 (0.59, 4.41) |
|  | 45-54 | 1.29 (0.50, 3.31) | 1.25 (0.47, 3.35) |
|  | 55+ | 0.93 (0.35, 2.46) | 0.94 (0.34, 2.64) |
| Race and Ethnicity | |  |  |
|  | Non-Hispanic White | Ref | Ref |
|  | Black or African American | 1.05 (0.50, 2.20) | 1.27 (0.59, 2.73) |
|  | Hispanic or Latinx | 1.12 (0.56, 2.22) | 1.24 (0.61, 2.55) |
|  | Mixed Race or Other | 1.71 (0.97, 3.03) | 1.79 (0.99, 3.23) |
| Gender Identity | |  |  |
|  | Cisgender Male | Ref | Ref |
|  | Cisgender Female | 0.95 (0.56, 1.62) | 1.15 (0.66, 2.02) |
|  | Transgender, Nonbinary, or Other | 1.31 (0.45, 3.82) | 1.74 (0.54, 5.56) |
| Duration Homeless | |  |  |
|  | <6 months | Ref | Ref |
|  | 6-12 months | 4.44 (1.14, 17.27)* | 3.87 (0.95, 15.68) |
|  | 1-2 years | 3.94 (1.11, 14.05)* | 3.47 (0.93, 12.98) |
|  | >2 years | 4.50 (1.34, 15.12)* | 3.72 (1.05, 13.12)* |
| Primary Sleeping Setting | |  |  |
|  | Sheltered or other | Ref | Ref |
|  | Outside, in a car, or an abandoned building | 2.04 (1.18, 3.53)* | 1.73 (0.96, 3.14) |

** indicates statistical significance at the alpha=0.05 level.*

**Supplemental Table 5.** **Unadjusted and adjusted odds ratios of the association between involuntary displacement and chronic health outcomes among a cross-sectional sample of people experiencing homelessness in Denver, Colorado, September 2018-February 2019.**

| **Characteristic** | | **Unadjusted OR (95% CI)** | **Adjusted OR (95% CI)** |
| --- | --- | --- | --- |
| Involuntary Displacement in Past 12 Months | |  |  |
|  | Never | Ref | Ref |
|  | 1 or more times | 0.95 (0.58, 1.56) | 1.08 (0.63, 1.86) |
| Age | |  |  |
|  | Under 24 | Ref | Ref |
|  | 25-34 | 0.73 (0.22, 2.41) | 0.72 (0.22, 2.42) |
|  | 35-44 | 1.30 (0.43, 3.96) | 1.37 (0.44, 4.28) |
|  | 45-54 | 2.75 (0.98, 7.72) | 2.83 (0.99, 8.16) |
|  | 55+ | 2.51 (0.89, 7.10) | 2.58 (0.88, 7.59) |
| Race and Ethnicity | |  |  |
|  | Non-Hispanic White | Ref | Ref |
|  | Black or African American | 0.98 (0.45, 2.09) | 0.88 (0.40, 1.95) |
|  | Hispanic or Latinx | 1.16 (0.58, 2.32) | 1.12 (0.54, 2.32) |
|  | Mixed Race or Other | 1.40 (0.78, 2.53) | 1.24 (0.73, 2.47) |
| Gender Identity | |  |  |
|  | Cisgender Male | Ref | Ref |
|  | Cisgender Female | 1.81 (1.07, 3.04)* | 1.74 (1.00, 3.01) |
|  | Transgender, Nonbinary, or Other | 1.32 (0.41, 4.17) | 1.70 (0.50, 5.85) |
| Duration Homeless | |  |  |
|  | <6 months | Ref | Ref |
|  | 6-12 months | 0.97 (0.37, 2.55) | 1.22 (0.43, 3.44) |
|  | 1-2 years | 0.61 (0.26, 1.46) | 0.84 (0.33, 2.17) |
|  | >2 years | 0.76 (0.35, 1.62) | 0.87 (0.37, 2.04) |
| Primary Sleeping Setting | |  |  |
|  | Sheltered or other | Ref | Ref |
|  | Outside, in a car, or an abandoned building | 0.922 (0.55, 1.53) | 1.06 (0.60, 1.87) |

** indicates statistical significance at the alpha=0.05 level.*

**Supplemental Table 6. Unadjusted and adjusted odds ratios of the association between involuntary displacement and musculoskeletal and disability outcomes among a cross-sectional sample of people experiencing homelessness in Denver, Colorado, September 2018-February 2019.**

| **Characteristic** | | **Unadjusted OR (95% CI)** | **Adjusted OR (95% CI)** |
| --- | --- | --- | --- |
| Involuntary Displacement in Past 12 Months | |  |  |
|  | Never | Ref | Ref |
|  | 1 or more times | 1.42 (0.95, 2.14) | 1.33 (0.85, 2.09) |
| Age | |  |  |
|  | Under 24 | Ref | Ref |
|  | 25-34 | 0.81 (0.37, 1.76) | 0.75 (0.33, 1.67) |
|  | 35-44 | 1.19 (0.54, 2.60) | 1.15 (0.51, 2.60) |
|  | 45-54 | 1.25 (0.59, 2.66) | 1.30 (0.59, 2.85) |
|  | 55+ | 1.59 (0.74, 3.42) | 1.76 (0.78, 3.94) |
| Race and Ethnicity | |  |  |
|  | Non-Hispanic White | Ref | Ref |
|  | Black or African American | 0.70 (0.38, 1.27) | 0.68 (0.37, 1.28) |
|  | Hispanic or Latinx | 0.79 (0.45, 1.40) | 0.80 (0.44, 1.44) |
|  | Mixed Race or Other | 1.02 (0.62, 1.70) | 1.01 (0.60, 1.70) |
| Gender Identity | |  |  |
|  | Cisgender Male | Ref | Ref |
|  | Cisgender Female | 1.48 (0.93, 2.35) | 1.67 (1.02, 2.72)* |
|  | Transgender, Nonbinary, or Other | 1.56 (0.57, 4.39) | 2.31 (0.80, 6.68) |
| Duration Homeless | |  |  |
|  | <6 months | Ref | Ref |
|  | 6-12 months | 1.83 (0.78, 4.32) | 1.97 (0.80, 4.88) |
|  | 1-2 years | 1.49 (0.72, 3.08) | 1.67 (0.76, 3.65) |
|  | >2 years | 1.61 (0.83, 3.10) | 1.50 (0.73, 3.09) |
| Primary Sleeping Setting | |  |  |
|  | Sheltered or other | Ref | Ref |
|  | Outside, in a car, or an abandoned building | 1.57 (1.03, 2.40)* | 0.84 (0.55, 1.30) |

** indicates statistical significance at the alpha=0.05 level.*

**Supplemental Table 7.** **Unadjusted and adjusted odds ratios of the association between involuntary displacement and chronic mental and emotional outcomes among a cross-sectional sample of people experiencing homelessness in Denver, Colorado, September 2018-February 2019.**

| **Characteristic** | | **Unadjusted OR (95% CI)** | **Adjusted OR (95% CI)** |
| --- | --- | --- | --- |
| Involuntary Displacement in Past 12 Months | |  |  |
|  | Never | Ref | Ref |
|  | 1 or more times | 0.84 (0.55, 1.30) | 0.89 (0.56, 1.43) |
| Age | |  |  |
|  | Under 24 | Ref | Ref |
|  | 25-34 | 0.58 (0.24, 1.39) | 0.59 (0.24, 1.43) |
|  | 35-44 | 0.76 (0.31, 1.84) | 0.74 (0.29, 1.84) |
|  | 45-54 | 0.62 (0.26, 1.46) | 0.60 (0.25, 1.44) |
|  | 55+ | 0.64 (0.27, 1.51) | 0.60 (0.24, 1.46) |
| Race and Ethnicity | |  |  |
|  | Non-Hispanic White | Ref | Ref |
|  | Black or African American | 0.79 (0.42, 1.49) | 0.79 (0.41, 1.52) |
|  | Hispanic or Latinx | 0.88 (0.48, 1.62) | 0.88 (0.47, 1.63) |
|  | Mixed Race or Other | 0.74 (0.44, 1.26) | 0.75 (0.44, 1.27) |
| Gender Identity | |  |  |
|  | Cisgender Male | Ref | Ref |
|  | Cisgender Female | 1.22 (0.75, 1.98) | 1.17 (0.71, 1.93) |
|  | Transgender, Nonbinary, or Other | 0.60 (0.23, 1.57) | 0.52 (0.19, 1.43) |
| Duration Homeless | |  |  |
|  | <6 months | Ref | Ref |
|  | 6-12 months | 0.82 (0.32, 2.10) | 0.82 (0.31, 2.19) |
|  | 1-2 years | 0.75 (0.33, 1.69) | 0.70 (0.29, 1.65) |
|  | >2 years | 0.68 (0.33, 1.43) | 0.70 (0.32, 1.56) |
| Primary Sleeping Setting | |  |  |
|  | Sheltered or other | Ref | Ref |
|  | Outside, in a car, or an abandoned building | 0.84 (0.54, 1.33) | 0.92 (0.56, 1.50) |

** indicates statistical significance at the alpha=0.05 level.*

**Supplemental Table 8.** **Unadjusted and adjusted odds ratios of the association between involuntary displacement and acute worsening mental health among a cross-sectional sample of people experiencing homelessness in Denver, Colorado, September 2018-February 2019.**

| **Characteristic** | | **Unadjusted OR (95% CI)** | **Adjusted OR (95% CI)** |
| --- | --- | --- | --- |
| Involuntary Displacement in Past 12 Months | |  |  |
|  | Never | Ref | Ref |
|  | 1 or more times | 1.97 (1.26, 3.07)* | 2.00 (1.24, 3.24)* |
| Age | |  |  |
|  | Under 24 | Ref | Ref |
|  | 25-34 | 0.98 (0.43, 2.27) | 1.03 (0.43, 2.45) |
|  | 35-44 | 1.87 (0.82, 4.27) | 2.05 (0.86, 4.87) |
|  | 45-54 | 1.01 (0.45, 2.27) | 1.08 (0.47, 2.52) |
|  | 55+ | 1.29 (0.58, 2.89) | 1.55 (0.66, 3.64) |
| Race and Ethnicity | |  |  |
|  | Non-Hispanic White | Ref | Ref |
|  | Black or African American | 1.12 (0.60, 2.08) | 1.21 (0.64, 2.31) |
|  | Hispanic or Latinx | 0.74 (0.40, 1.38) | 0.83 (0.44, 1.58) |
|  | Mixed Race or Other | 1.10 (0.65, 1.85) | 1.14 (0.66, 1.96) |
| Gender Identity | |  |  |
|  | Cisgender Male | Ref | Ref |
|  | Cisgender Female | 1.34 (0.84, 2.14) | 1.44 (0.87, 2.36) |
|  | Transgender, Nonbinary, or Other | 0.71 (0.24, 2.04) | 0.86 (0.28, 2.61) |
| Duration Homeless | |  |  |
|  | <6 months | Ref | Ref |
|  | 6-12 months | 1.12 (0.44, 2.81) | 0.94 (0.36, 2.46) |
|  | 1-2 years | 1.60 (0.74, 3.50) | 1.35 (0.59, 3.12) |
|  | >2 years | 1.20 (0.59, 2.45) | 0.91 (0.42, 1.99) |
| Primary Sleeping Setting | |  |  |
|  | Sheltered or other | Ref | Ref |
|  | Outside, in a car, or an abandoned building | 1.30 (0.83, 2.03) | 1.18 (0.72, 1.94) |

** indicates statistical significance at the alpha=0.05 level.*
